# Three-dimensional modeling of single stranded DNA hairpins for aptamer-based biosensors

**DOI:** 10.1038/s41598-017-01348-5

**Published:** 2017-04-26

**Authors:** Iman Jeddi, Leonor Saiz

**Affiliations:** Modeling of Biological Networks and Systems Therapeutics Laboratory, Department of Biomedical Engineering, University of California, 451 East Health Sciences Drive, Davis, CA 95616 USA

## Abstract

Aptamers consist of short oligonucleotides that bind specific targets. They provide advantages over antibodies, including robustness, low cost, and reusability. Their chemical structure allows the insertion of reporter molecules and surface-binding agents in specific locations, which have been recently exploited for the development of aptamer-based biosensors and direct detection strategies. Mainstream use of these devices, however, still requires significant improvements in optimization for consistency and reproducibility. DNA aptamers are more stable than their RNA counterparts for biomedical applications but have the disadvantage of lacking the wide array of computational tools for RNA structural prediction. Here, we present the first approach to predict from sequence the three-dimensional structures of single stranded (ss) DNA required for aptamer applications, focusing explicitly on ssDNA hairpins. The approach consists of a pipeline that integrates sequentially building ssDNA secondary structure from sequence, constructing equivalent 3D ssRNA models, transforming the 3D ssRNA models into ssDNA 3D structures, and refining the resulting ssDNA 3D structures. Through this pipeline, our approach faithfully predicts the representative structures available in the Nucleic Acid Database and Protein Data Bank databases. Our results, thus, open up a much-needed avenue for integrating DNA in the computational analysis and design of aptamer-based biosensors.

## Introduction

Cellular-level protein production is traditionally determined using several bioanalytical approaches, which rely on antibody or enzyme recognition. These include flow cytometry coupled with intracellular cytokine staining, enzyme-linked immunospot (ELISPOT), enzyme linked immunosorbent assay (ELISA), and polymerase chain reaction (PCR)^1, 2^. ELISA and PCR are robust technologies for detecting either cytokines or cytokine mRNA in blood; however, they cannot be used to identify specific populations of cytokine producing cells^2^. In contrast, flow cytometry and ELISPOT report the frequency of cytokine positive cells but not the cytokine concentration^1^. While these well-established methods can be sensitive and robust, they employ complex detection procedures, involving expensive reagents and multiple time consuming washing steps. In addition, these traditional strategies provide no information about the temporal dynamics of cytokine production, which is vital information in understanding the body’s immune response^3^. In order to fill this gap, aptamer-based affinity sensing strategies are emerging as viable alternatives to antibody-based immunoassays^4^.

Aptamer-based biorecognition elements are short nucleic acid molecules and thus are more robust and simple than antibody-based probes. Aptamers bind specifically diverse targets including ions, organic dyes, amino acids, nucleotides, RNA, biological cofactors, other small organic molecules, oligosaccharides, peptides, toxins, enzymes, growth factors, transcription factors, antibodies, viral proteins and/or components, cells, and bacteria^5^. The selection of aptamers is termed Systematic Evolution of Ligands by Exponential Enrichment (SELEX) and involves the discovery of full-length aptamers from large pools of randomized single-stranded DNA or RNA (10^14^ to 10^15^ variants). The selection process is highly iterative and involves exposing the oligonucleotides to a target that is either coupled to a matrix or surface. The unbound molecules are then washed away and the bound molecules are recovered and amplified. This process results in highly robust and specific aptamers that are 25–40 nucleotides long^6^.

The relatively simple chemical structure of aptamers allows the insertion of electrochemical or fluorescent reporter molecules^7^ as well as surface-binding agents^8^ in specific locations on the oligonucleotide^9^. During probe-target binding, the conformation change of the aptamer may be exploited to generate an analytical signal^10^. A number of aptamer-based biosensors have been successfully used to measure cell secretion of proteins^11, 12^; however, several opportunities for improvement remain before commercialization is feasible^13^. These include enhancing the sensitivity and improving the manufacturability and repeatability of aptamer-based sensors. Both of these challenges are not easily overcome without a better understanding of the molecular level interactions of the aptamer-biosensor surface (for improving manufacturability, reproducibility, and sensitivity) as well as of the aptamer-protein complex (for improving specificity and sensitivity).

Despite the growing widespread use in biotechnology and the substantial number of biomedical applications of DNA aptamers, including its clinical use as therapeutic agents for a number of human diseases^14–16^ and its increasing use in drug delivery^17^, the considerable array of computational tools available for single stranded RNA structure prediction (e.g. see refs 18–21 for recent reviews)^18–21^ are lacking for its DNA counterpart. Until now, the 3D computational tools available for DNA have been restricted to model mainly double-stranded DNA structures^22^, lacking the ability to analyze single-stranded DNA hairpins and other more complex structures. The ability to faithfully predict the 3D structure of single stranded DNA and RNA from sequence has the potential to revolutionize the way aptamers are selected and allow for crucial applications not only into aptamer design but also for biosensor set-up design and the molecular level understanding of structure and dynamics of single stranded oligonucleotide systems^23^.

Here, we present the first approach to predict the three-dimensional structures of single stranded DNA required for aptamer applications that extends current sequence-based computational efforts for RNA^18–21^. Our approach faithfully predicts the representative resolved structures available in the Nucleic Acid Database (NDB) and Protein Data Bank (PDB) databases from a pipeline that integrates 2D and 3D structural tools, including Mfold, Assemble 2, Chimera, VMD, and Molecular Dynamics (MD) simulations. Explicitly, we build ssDNA secondary structure from sequence, construct equivalent 3D ssRNA models, transform the 3D ssRNA models into ssDNA 3D structures, and finally refine the resulting ssDNA 3D structures through energy minimization. To thoroughly evaluate our approach, we considered all hairpin-like ssDNA molecules with experimentally solved 3D structures selected through an exhaustive search for ssDNA molecules and aptamers in the PDB database. Our results indicate that this approach works exceptionally well for the hairpin-like structural motif of ssDNA, the focus of the current work. To test the robustness of the results, we performed additional atomistic MD simulations for a sub-set of representative ssDNA molecules and aptamers. The atomistic details available in MD simulations with explicit solvent have been fundamental at uncovering the molecular level mechanisms of key experimental observations and deepen our understanding of the interactions and properties of biological complexes in their natural environment^24–28^. Partly because of the lack of solved 3D structures, very few MD simulation studies have focused on aptamers^29, 30^. Our results show that MD simulations can, indeed, be used to further improve the structural predictions and that the predictions are representative of those obtained from the dynamics of the systems under conditions that mimic their targeted environment.

## Methods

### Workflow for three-dimensional structure generation from sequence

**Figure 1:**
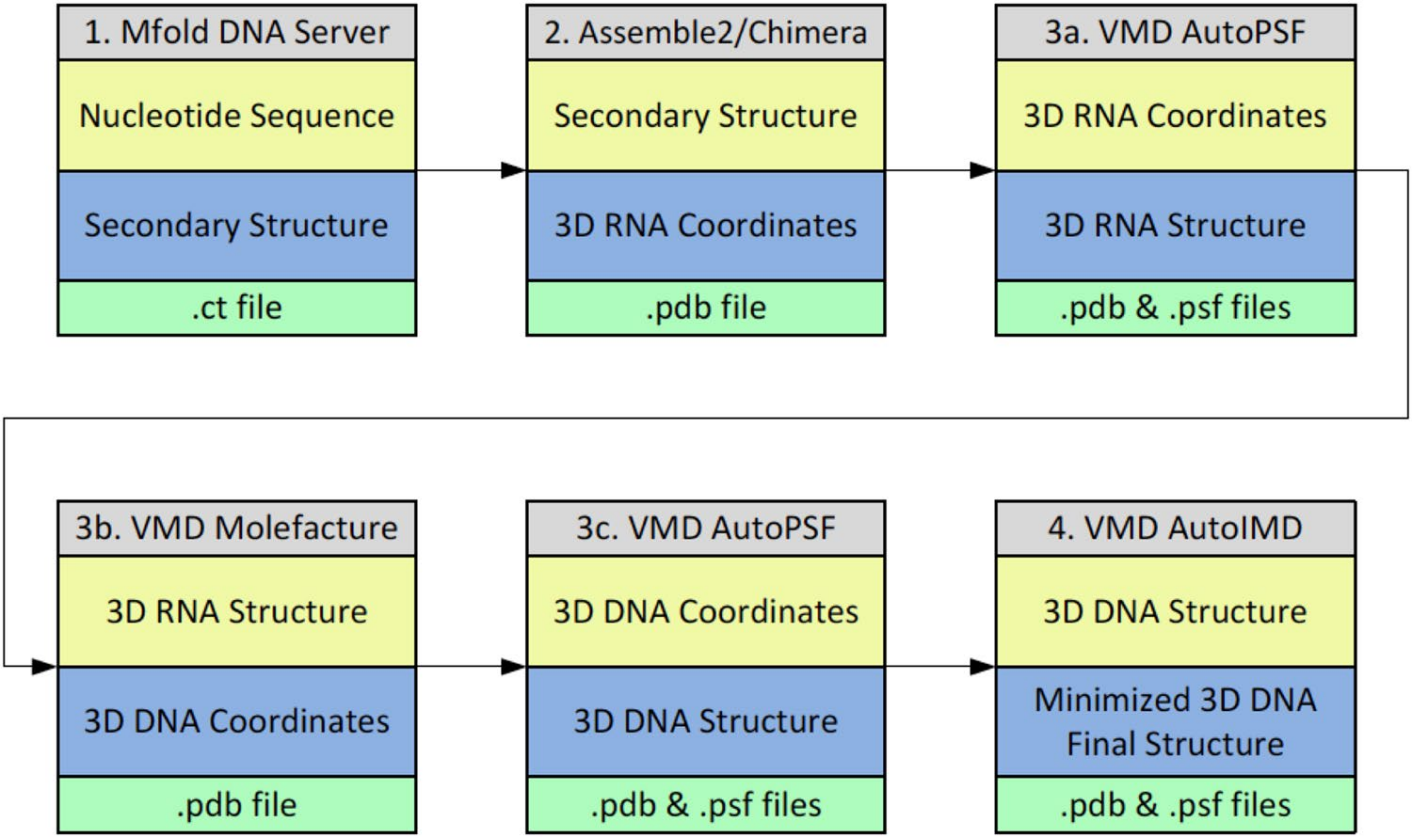
Workflow used to construct the ssDNA 3D structures from the sequence. The approach consists of four main steps, involving building the ssDNA secondary structure from the sequence using Mfold (step 1), constructing refined equivalent 3D ssRNA models using Assemble2/Chimera (step 2), translating the 3D ssRNA models into ssDNA models using VMD (step 3), and refining the 3D ssDNA structures using VMD (step 4).

The workflow to construct the 3D structures of the ssDNA molecules from the nucleotide sequence consists of four main steps (Fig. 1), comprising building the DNA secondary structure using Mfold in step 1, constructing refined equivalent 3D RNA models using Assemble2 and Chimera in step 2, translating the 3D RNA models into DNA models using VMD in step 3, and refining the final 3D DNA structure through minimization using VMD in step 4, as described below. Typically, this process takes about one hour to complete. Illustrative examples of the structures obtained at the end of each of the four steps of the workflow are shown in Supplementary Figure 1.

#### Step 1: Build ssDNA secondary structure from sequence

The first step of our approach is the prediction of secondary structures from DNA sequences. The input consists of the DNA sequences, which for this study were selected after searching the PDB database with a focus on the hairpin-like common fold of ssDNA, as detailed in the results section. Starting with the nucleotide sequence, the secondary structures of the ssDNA molecules were predicted using the mfold web server (http://mfold.rna.albany.edu/?=mfold)^31^, based on free energy minimization techniques. In mfold, all possible secondary structures are approximated based on Watson-Crick base pairing and the most thermodynamically stable structures are selected^32, 33^. The initial sequences were selected as linear at a temperature of 37 °C and ionic concentration of 1 M of Na^+^, 0 M of Mg^2+^, computing only fold configurations within 5% from the minimum free energy, and considering a maximum number of 50 folds with no limit to the maximum distance between paired bases. In addition to the predicted secondary structure of the ssDNA this step provides the minimum free energy of the fold.

#### Step 2: Construct equivalent 3D ssRNA models and refine structures

The second step is to use the predicted secondary structures as a starting point to generate the 3D structures of the equivalent ssRNA models using Assemble2/Chimera. The 3D structures were modeled and visualized using Assemble2^34^ and Chimera^35^ following a manual process of individually selecting the 2D helical and non-helical elements of the ssDNA molecule and translating the residues into equivalent 3D RNA models. The 3D RNA models were then refined using 100 iterations to remove geometric deficiencies and optimize the structural parameters such as bond length and angles, planarity of certain groups, non-bonded contacts, and restricted torsion angles. The refinement was achieved by the geometrical least squares method using the Konnert–Hendrickson algorithm^36^ as implemented in the Assemble2 program.

#### Step 3: Transform the 3D ssRNA models into ssDNA 3D structures

In the third step, the refined ssRNA 3D structures were imported into VMD^37^, where hydrogen atoms were added using the AutoPSF VMD plugin (*step 3a*), and the ssRNAs modified into ssDNA 3D structures by: (i) identifying each uracil residue and replacing the H5 atom with a methyl group using the Molefacture VMD plugin (*step 3b*) and (ii) replacing the ribose sugar backbone with deoxyribose using the AutoPSF VMD plugin and manually renaming the modified uracil residues to thymine in the pdb file (*step 3c*). The output of this step consists of both pdb and psf files that contain the atomic coordinates (.pdb) and atom type, charge, mass and bonding information (.psf).

#### Step 4: Refine final ssDNA 3D structures

The fourth step of our approach consists of further refinement of the ssDNA 3D structures obtained in step 3. It uses the AutoIMD VMD plugin to refine the structures through energy minimization using 10,000 iterations. The output of this step consists of the final coordinate (.pdb) file in addition to the psf file obtained in the previous step. We will use these two files, in addition to files containing the topology of the molecules and the force-field parameters, for conducting further analysis through molecular dynamics simulations as detailed below.

### Molecular Dynamics Simulations

#### Description of the systems and initial structure set-up

We carried out molecular dynamics simulation studies for 5 ssDNA sequences from the pool of 24 structures selected after an exhaustive search from the PDB database, as detailed in the results section, using as initial configurations our predicted 3D structure and the corresponding experimentally resolved structure from the PDB database. For the sake of simplicity, throughout this paper, the five original ssDNA resolved through conventional experimental methods will be referred to as “original” and those derived here using computational modeling methods will be referred to as “predicted”. They are labeled using their corresponding PDB IDs. The predicted and original structures for the single stranded DNA hairpins corresponding to the PDB ID entries 1BJH, 1LA8, 2M8Y, 2VAH, and 2L5K were each solvated in a water box to closer represent the biological as well as the biosensor environment. In the case of the original systems, first we used the AutoPSF VMD plugin to add the hydrogen atoms to the ssDNAs, missing from the NMR solution structures. Using the Solvate plugin in VMD^37^, a layer of water of about 25 Å in each direction from the atom with the largest coordinate in that direction was created to fully immerse the ssDNA molecules. In addition, each system was neutralized by replacing a predetermined number of water molecules with sodium ions. Table 1 contains the resulting dimensions of the simulation cells as well as the type and number of ions used to neutralize each system.

**Table 1:** Dimensions of the simulation box, number of atoms of the ssDNA molecules, ion type and number of ions in the system, and total number of atoms for each of the 10 different molecular dynamics simulations.

Structure	*L*_X_ (Å)	*L*_Y_ (Å)	*L*_Z_ (Å)	ssDNA atoms	Ion Type	# of Ions	Total Atoms
1BJH original	70	74	71	349	Na^+^	10	32780
1BJH predicted	75	73	70	349	Na^+^	10	33947
1LA8 original	72	79	77	411	Na^+^	12	39720
1LA8 predicted	72	72	78	411	Na^+^	12	36867
2M8Y original	74	77	78	474	Na^+^	14	39899
2M8Y predicted	74	71	81	474	Na^+^	14	38411
2VAH original	72	74	82	569	Na^+^	17	38974
2VAH predicted	74	74	87	569	Na^+^	17	42901
2L5K original	75	75	94	728	Na^+^	22	47412
2L5K predicted	75	75	94	728	Na^+^	22	46119

#### Molecular Dynamics Simulation Details

We carried out the ten different simulations using the atomistic molecular dynamics (MD) simulation code NAMD^38^. The details of each system are summarized in Table 1. All the MD simulations were carried out using the NAMD2.9 software package with the recent version of the all-atom CHARMM force field for the nucleic acids^39^ (CHARMM27) and the rigid TIP3P model for water^40^, which is consistent with the nucleic acids force field. Following standard procedures, the solvated ssDNA systems were first minimized for 100,000 steps using the conjugate gradient energy minimization method as implemented in NAMD. This was followed by a NVT equilibration run at a temperature of 300 K and a NPT production run of a total of 10 ns at a temperature of 300 K and a pressure of 1 atm. To maintain these conditions, we used the Langevin dynamics method with a friction constant of 1 ${{\rm{p}}{\rm{s}}}^{-1}$ and the Nose-Hoover Langevin piston method^41^. The simulations were carried out using a time step of 2 fs. In all the simulations, we used three-dimensional periodic boundary conditions and the minimum image convention^42^ to calculate the short-range Lennard-Jones interactions using a spherical cutoff distance of 12 Å with a switch distance of 10 Å. The long-range electrostatic interactions were calculated by using the particle-mesh Ewald (PME) method^43^.

## Results and Discussion

### Selection of ssDNA Candidates

We performed an exhaustive search for ssDNA molecules and aptamers with experimentally solved 3D structures in the Protein Data Bank database (http://www.pdb.org). The selection method consisted of two independent searches using the following keywords: “DNA hairpin” and “aptamer DNA”. This resulted in a total of 772 entries. The search results were then filtered by ‘Polymer Type’; only including entries that were classified under “DNA”. This resulted in reducing the pool of potential candidates to 97 entries as detailed in Table 2. Subsequently, 20 entries containing ligands were excluded from the list of candidates. The remaining 77 structures were then individually reviewed and 53 additional structures that did not correspond to the hairpin-like structural motif were eliminated. The exclusion criteria in this last step eliminated the following types of structures: duplex, triplex, three-way junction, dimer, dimer G-quadruplex, quadruplex duplex hybrid, complexed with non-protein ligand, Z-DNA, and non-nucleotide sequence modifications. This last step resulted in the exclusion from the list of all the DNA sequences with a G-score greater than zero as calculated using the QGRS Mapper software program^44^. QGRS Mapper uses a scoring system to predict the presence of quadruplex forming G-rich sequences in nucleotide sequences and non-zero scores indicate the possibility of G-quadruplex formation with higher scores representing better G-quadruplex forming candidates. The remaining pool of 24 candidates (Table 2), with 21 different sequences, was selected as our initial set of 21 DNA sequences for 3D structural prediction.

**Table 2:** Protein Data Bank database search results for the selection of ssDNA candidates.

PMID	PDB ID	Chain Length	Sequence	QGRS	Exclude	Exclusion Criteria
8069626	134D	31	TCCTCCTTTTTTAGGAGGATTTTTTGGTGGT	15	Yes	Triplex
8069626	135D	31	TCCTCCTTTTTTAGGAGGATTTTTTGGTGGT	15	Yes	Triplex
8069626	136D	31	TCCTCCTTTTTTAGGAGGATTTTTTGGTGGT	15	Yes	Triplex
7613864	184D	7	GCATGCT	0	Yes	Dimer Quadruplex
9737926	1A8N	12	GGGCTTTTGGGC	0	Yes	Dimer Quadruplex
9737927	1A8W	12	GGGCTTTTGGGC	0	Yes	Dimer Quadruplex
9092659	1AC7	16	ATCCTAGTTATAGGAT	0	No	N/A
9398169	1AO9	13	GAGAGAXTCTCTC	0	Yes	Complexed with non-protein ligand
7664126	1AU6	8	CATGCATG	0	Yes	Complexed with non-protein ligand
9384529	1AW4	27	ACCTGGGGGAGTATTGCGGAGGAAGGT	0	Yes	Complexed with non-protein ligand
**9000625**	**1BJH**	**11**	**GTACAAAGTAC**	**0**	**No**	**N/A**
9367776	1C11	11	TCCCGTTTCCA	0	Yes	Dimer Quadruplex
12371856	1CS7	13	GUTTTGXCAAAAC	0	Yes	Complexed with non-protein ligand
2299669	1D16	16	CGCGCGTTTTCGCGCG	0	Yes	Z-DNA
10653638	1DB6	22	CGACCAACGTGTCGCCTGGTCG	0	Yes	Complexed with non-protein ligand
10756190	1DGO	18	AGGATCCTUTTGGATCCT	0	No	N/A
N/A	1ECU	19	GCGCGAAACTGTTTCGCGC	0	No	N/A
10924101	1EN1	18	GTCCCTGTTCGGGCGCCA	0	No	N/A
11090280	1EZN	36	CGTGCACCCGCTTGCGGCGACTTGTCGTTGTGCACG	0	Yes	Three way junction
11790144	1FV8	11	TATCATCGATA	0	Yes	Non-nucleotide modified residues
11352724	1G5L	6	CCAAAG	0	Yes	Complexed with non-protein ligand
11352724	1GJ2	6	CCAAAG	0	Yes	Complexed with non-protein ligand
11952790	1IDX	18	AGGATCCTTUTGGATCCT	0	No	N/A
11952790	1II1	18	AGGATCCUTTTGGATCCT	0	No	N/A
11843626	1JU0	23	CTTGCTGAAGCGCGCACGGCAAG	0	Yes	Dimer
11843626	1JUA	23	CTTGCTGAAGCGCGCACGGCAAG	0	Yes	Dimer
11991355	1JVE	27	CCTAATTATAACGAAGTTATAATTAGG	0	No	N/A
12449414	1KR8	7	GCGAAGC	0	No	N/A
11895443	1L0R	14	ACGAAGTGCGAAGC	0	Yes	Complexed with non-protein ligand
**11849039**	**1LA8**	**13**	**CGCGGTGTCCGCG**	**0**	**No**	**N/A**
11849039	1LAE	13	CGCGGTPTCCGCG	0	Yes	Non-nucleotide modified residues
11849038	1LAI	13	CGCGGTGTCCGCG	0	Yes	Duplex
11849038	1LAQ	13	CGCGGTPTCCGCG	0	Yes	Duplex
11849038	1LAS	13	CGCGGTPTCCGCG	0	Yes	Duplex
12560479	1MF5	7	GCATGCT	0	Yes	Dimer Quadruplex
12564921	1MP7	10	GCCAGAGAGC	0	Yes	Complexed with non-protein ligand
12755609	1NGO	27	CTCTTTTTGTAAGAAATACAAGGAGAG	0	No	N/A
12755609	1NGU	27	CTCTCCTTGTATTTCTTACAAAAAGAG	0	No	N/A
12758081	1P0U	13	GCATCGACGATGC	0	No	N/A
8525381	1PNN	24	GAAGAAGAG	0	Yes	Triplex
12449414	1PQT	7	GCGAAGC	0	No	N/A
12952463	1PUY	13	GTTTTGXCAAAAC	0	Yes	Complexed with non-protein ligand
10481034	1QE7	22	CTAGAGGATCCTTTUGGATCCT	0	No	N/A
N/A	1QYK	7	GCATGCT	0	Yes	Dimer Quadruplex
N/A	1QYL	7	GCATGCT	0	Yes	Dimer Quadruplex
15199171	1SNJ	36	CGTGCAGCGGCTTGCCGGCACTTGTGCTTCTGCACG	0	Yes	Three way junction
14684897	1UE2	9	GCGAAAGCT	0	Yes	Duplex
14684897	1UE3	8	GCGAAAGC	0	Yes	Duplex
8901550	1XUE	17	GTGGAATGCAATGGAAC	0	No	N/A
7583654	1ZHU	10	CAATGCAATG	0	No	N/A
8548453	229D	17	CCAGACUGAAGAUCUGG	0	Yes	Non-nucleotide modified residues
9818148	2ARG	30	TGACCAGGGCAAACGGTAGGTGAGTGGTCA	18	Yes	Complexed with non-protein ligand
16620121	2AVH	11	GGGGTTTGGGG	0	Yes	G-Quadruplex
N/A	2F1Q	42	GCACTGCATCCTTGGACGCTTGCGCCACTTGTGGTGCAGTGC	0	Yes	Four way junction
16866556	2GKU	24	TTGGGTTAGGGTTAGGGTTAGGGA	42	Yes	G-Quadruplex
N/A	2K67	17	TTAATTTNNNAAATTAA	0	Yes	Non-nucleotide modified residues
N/A	2K68	17	TTAATTTNNNAAATTAA	0	Yes	Non-nucleotide modified residues
N/A	2K69	17	TTAATTTNNNAAATTAA	0	Yes	Non-nucleotide modified residues
19374420	2K71	8	GCGAAAGC	0	No	N/A
19321501	2K8Z	8	TCGTTGCT	0	Yes	Dimer
19070621	2KAZ	13	GGGACGTAGTGGG	0	Yes	Dimer Quadruplex
21410196	2L13	13	TATTATXATAATA	0	Yes	Non-nucleotide modified residues
**22129448**	**2L5K**	**23**	**CAGTTGATCCTTTGGATACCCTG**	**0**	**No**	**N/A**
22507054	2LO5	12	GGCCGCAGTGCC	0	No	N/A
22507054	2LO8	10	GCCGCAGTGC	0	No	N/A
22507054	2LOA	10	GCCGCAGTGC	0	Yes	Complexed with non-protein ligand
22798499	2LSC	12	CGCGAAUUCGCG	0	Yes	Complexed with non-protein ligand
**23794476**	**2M8Y**	**15**	**CGCGAAGCATTCGCG**	**0**	**No**	**N/A**
23794476	2M8Z	27	GGTTGGCGCGAAGCATTCGCGGGTTGG	9	Yes	Quadruplex Duplex Hybrid
23794476	2M90	32	GCGCGAAGCATTCGCGGGGAGGTGGGGAAGGG	21	Yes	Quadruplex Duplex Hybrid
23794476	2M91	30	GGGAAGGGCGCGAAGCATTCGCGAGGTAGG	7	Yes	Quadruplex Duplex Hybrid
23794476	2M92	34	AGGGTGGGTGCTGGGGCGCGAAGCATTCGCGAGG	17	Yes	Quadruplex Duplex Hybrid
23794476	2M93	32	TTGGGTGGGCGCGAAGCATTCGCGGGGTGGGT	29	Yes	Quadruplex Duplex Hybrid
8658168	2NEO	19	CCCGATGCXGCAATTCGGG	0	Yes	Complexed with non-protein ligand
17362008	2O3M	22	AGGGAGGGCGCTGGGAGGAGGG	39	Yes	G-Quadruplex
17388570	2OEY	25	CCATCGTCTACCTTTGGTAGGATGG	0	Yes	Complexed with non-protein ligand
9020982	2PIK	23	CACTCCTGGTTTTTCCAGGAGTG	0	Yes	Complexed with non-protein ligand
18515837	**2VAH**	**18**	**AGGATCCTUTTGGATCCT**	**0**	**No**	**N/A**
18515837	2VAI	18	AGGATCCTUTTGGATCCT	0	No	N/A
22287624	3QXR	22	AGGGAGGGCGCUGGGAGGAGGG	39	Yes	G-Quadruplex
N/A	3T86	7	GCATGCT	0	Yes	Complexed with non-protein ligand
22409313	4DKZ	12	CGCGAAXXCGCG	0	Yes	Non-nucleotide modified residues
8107090	148D	15	GGTTGGTGTGGTTGG	20	Yes	G-Quadruplex
9384529	1AW4	27	ACCTGGGGGAGTATTGCGGAGGAAGGT	14	Yes	Complexed with non-protein ligand
9799703	1BUB	15	GGTTGGTGTGGTTGG	20	Yes	G-Quadruplex
10756199	1C32	15	GGTTGGTGTGGTTGG	20	Yes	G-Quadruplex
10756199	1C34	15	GGTTGGTGTGGTTGG	20	Yes	G-Quadruplex
10756199	1C35	15	GGTTGGTGTGGTTGG	20	Yes	G-Quadruplex
10756199	1C38	15	GGTTGGTGTGGTTGG	20	Yes	G-Quadruplex
10653638	1DB6	22	CGACCAACGTGTCGCCTGGTCG	0	Yes	Complexed with non-protein ligand
8757801	1QDF	15	GGTTGGTGTGGTTGG	20	Yes	G-Quadruplex
8757801	1QDH	15	GGTTGGTGTGGTTGG	20	Yes	G-Quadruplex
15214802	1RDE	15	GGTTGGTGTGGTTGG	20	Yes	G-Quadruplex
15637158	1Y8D	16	GGGGTGGGAGGAGGGT	21	Yes	Dimer Quadruplex
9818148	2ARG	30	TGACCAGGGCAAACGGTAGGTGAGTGGTCA	18	Yes	Complexed with non-protein ligand
17145716	2IDN	15	GGTTGGTGTGGTTGG	20	Yes	G-Quadruplex
23935071	2M53	25	TGTGGGGGTGGACGGGCCGGGTAGA	21	Yes	G-Quadruplex

The details of the selection criteria are discussed in the text (see results section). For each experimentally resolved structure from the PDB database, denoted by its PDB ID, the table provides the corresponding publication denoted by its PubMed identifier (PMID), the sequence as provided in the PDB database, the chain length, the G-score denoted by QGRS obtained using the QGRS Mapper, and, if the DNA candidate was excluded from our list of potential candidates, the exclusion criteria.

The 24 ssDNA molecules had 21 unique sequences ranging in length from 7 to 27 nucleotides, all with structures solved by NMR spectroscopy, and comprising a wide variety of systems of biological and biomedical interest, ranging from models of biologically relevant sequences, such as those to which HIV-1 nucleocapsid protein binds during reverse transcription^45^ or those with uracil (a constituent of RNA) bases^46^, to DNA aptamers, such as those that bind Mucin 1, a cell-surface glycoprotein overexpressed in a number of cancers^47^.

In addition, five representative ssDNA hairpins (Table 2; entries in bold typesetting), with PDB IDs 1BJH^48^, 1LA8^49^, 2M8Y^50^, 2VAH^46^, and 2L5K^47^, were selected from the 24 candidates for additional analysis through atomistic molecular dynamics simulations. The length of these molecules ranges from 11 (1BJH) to 24 (2L5K) nucleotides.

### Three-dimensional Structure Prediction from Sequence

**Figure 2:**
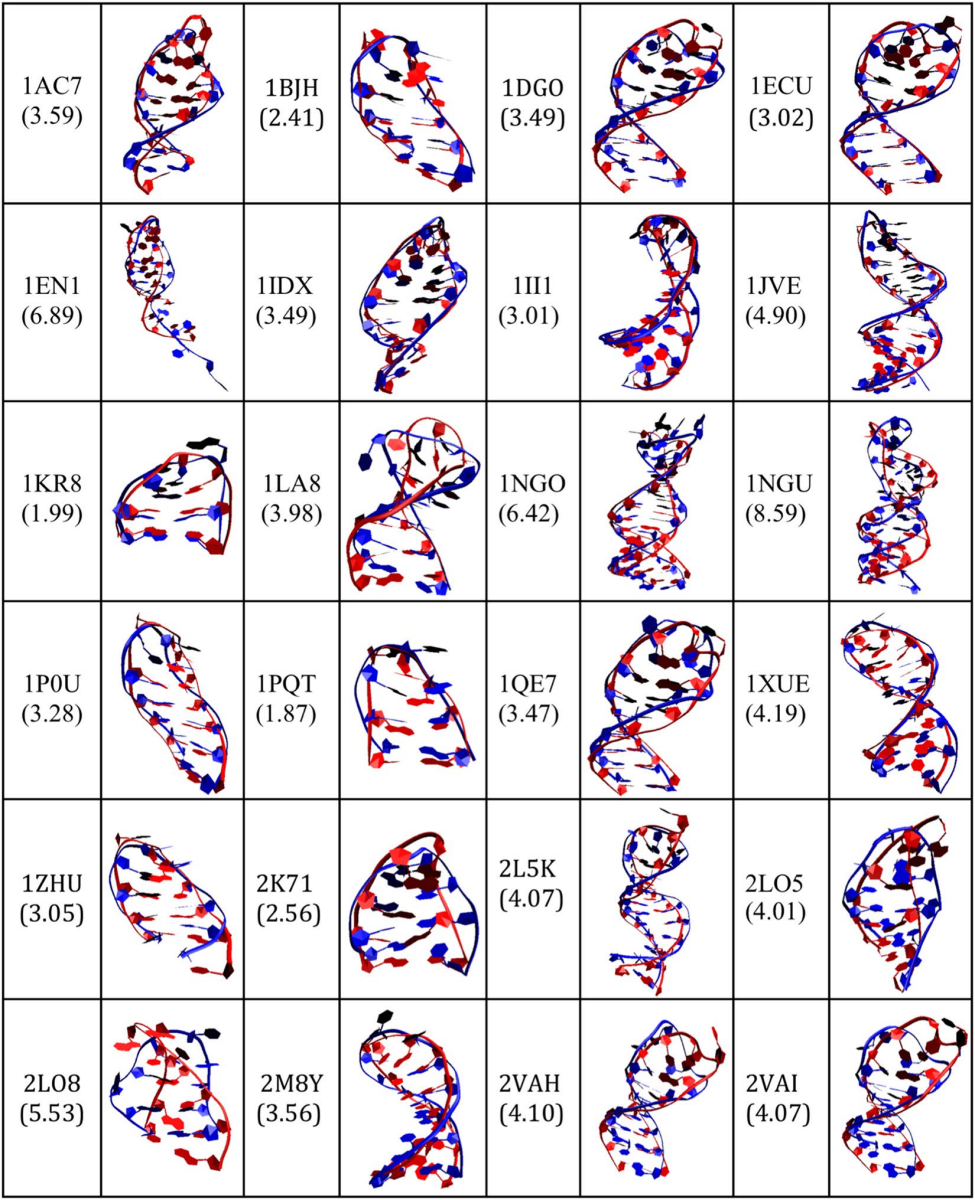
Overlay of the 3D predicted structures (ssDNA colored red) and the corresponding experimental structures downloaded from the PDB database (ssDNA colored blue) for the 24 ssDNA hairpin structures. Each structure is labeled by its corresponding PDB ID and the calculated RMSD values (in Angstroms) are shown in parenthesis.

**Figure 3:**
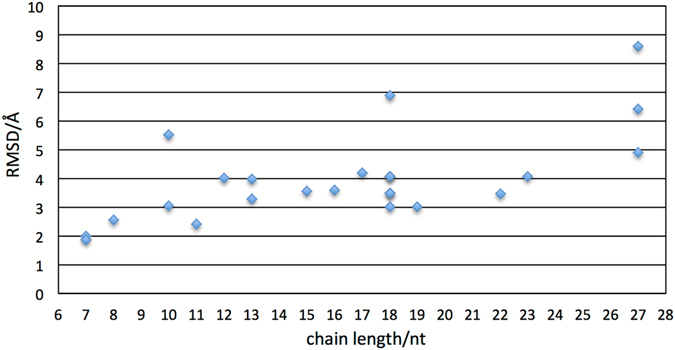
Values of the RMSD for the 24 ssDNA predicted structures with respect to the experimental ones as a function of the nucleotide chain length.

The approach followed, as indicated in the workflow (Fig. 1) and detailed in the methods section, successfully captures the 3D structures predicted from the 21 different sequences of the 24 ssDNA hairpins identified from the PDB database. In order to test the accuracy of the 3D prediction method, each predicted ssDNA structure was aligned with the corresponding ssDNA structure downloaded from the PDB database. To measure the degree of similarity, the corresponding Root Mean Square Deviation (RMSD) of the sugar-phosphate backbone between each pair of structures was calculated. Figure 2 shows an overlay of each of the 21 predicted 3D structures (ssDNA colored red) and the corresponding 24 NMR structures downloaded from the PDB database (ssDNA colored blue), together with calculated values of the RMSDs. Our results indicate that our approach is able to faithfully predict the 3D structure of a wide variety of ssDNA hairpins, with sequences ranging from 7 to 27 nucleotides. In all cases, the RMSD values obtained ranged from 1.9 Å (PDB ID: 1PQT) for the shortest sequences to 8.59 Å (1NGU) for the longest cases, and were typically around 3.5 Å, with an average value of 4 Å. Interestingly, our approach was also able to accurately predict the 3D structure for ssDNA sequences containing uracil bases, namely 1DGO, 1IDX, 1II1, 1QE7, 2VAH, and 2VAI. Three particular predicted structures, namely 2LO8, 1EN1, and 1NGU, differed more than those corresponding to typical values we obtained for other ssDNAs with similar sequence lengths. Figure 3 shows the RMSD values obtained for the 24 predicted structures with respect to the experimental ones as a function of chain length of the DNA. For instance, the RMSD obtained for 2LO8 with a chain length of 10 nucleotides is 5.53 Å, while the corresponding value for 1ZHU is 3.05 Å. Interestingly, the sequence 2LO8 is only 10nt, and is part of 2LO5. However, its RMSD is bigger than the latter because the two additional bases in 2LO5 make a CG base-pairing and increase the stability of the loop. In the case of 1EN1, the higher RMSD value (compared for instance with the value of 3.01 obtained for 1II1 with also 18 nucleotides) mostly originates from the unstructured tail at the 3’ end of the hairpin, which was predicted as more structured. In this case, additional molecular dynamics simulations, as the ones we present in the next section for five of the ssDNA sequences, are likely to improve the prediction of the model by relaxing the positions of the nucleotides at the tail.

The results obtained for the 3D predictions of the five ssDNA hairpins selected from the pool of 24 candidates for additional analysis through atomistic molecular dynamics simulations, namely 1BJH, 1LA8, 2M8Y, 2VAH, and 2L5K, were in good agreement with the experimental data. Specifically, we obtained RMSD values of ranging from ~2.4 Å for 1BJH with 11 nucleotides to ~4.1 Å for 2VAH and 2L5K with 18 and 23 nucleotides, respectively. In these five cases, the secondary structure prediction obtained in step 1 of our approach using Mfold as described in the methods section resulted in the following: 1BJH is characterized by a minimum free energy of 0.04 kcal/mol and consists of a 4-base pair stacked stem and a 3-nucleotide hairpin loop; 1LA8 is characterized by a minimum free energy of −4.9 kcal/mol and consists of a 5-base pair stacked stem and a 3-nucleotide hairpin loop; 2M8Y is characterized by a minimum free energy of −6.3 kcal/mol and consists of a 6-base pair helical stacked stem followed by a 3-nucleotide hairpin loop; 2VAH is characterized by a minimum free energy of −6.2 kcal/mol and consists of a 7-base pair helical stacked stem and a 4-nucleotide hairpin loop; and 2L5K is characterized by a minimum free energy of 0.05 kcal/mol and consists of a 3-base pair stacked stem attached to a 4-base pair stacked stem by two strings of 3-base single stranded nucleotides, followed by a 3-nucleotide hairpin loop. As in the other cases, the predicted secondary structures of these five ssDNA molecules are in agreement with the resolved structures available through the Protein Data Bank database.

### Refinement of 3D Predictions and Dynamics through Molecular Dynamics Simulations

**Figure 4:**
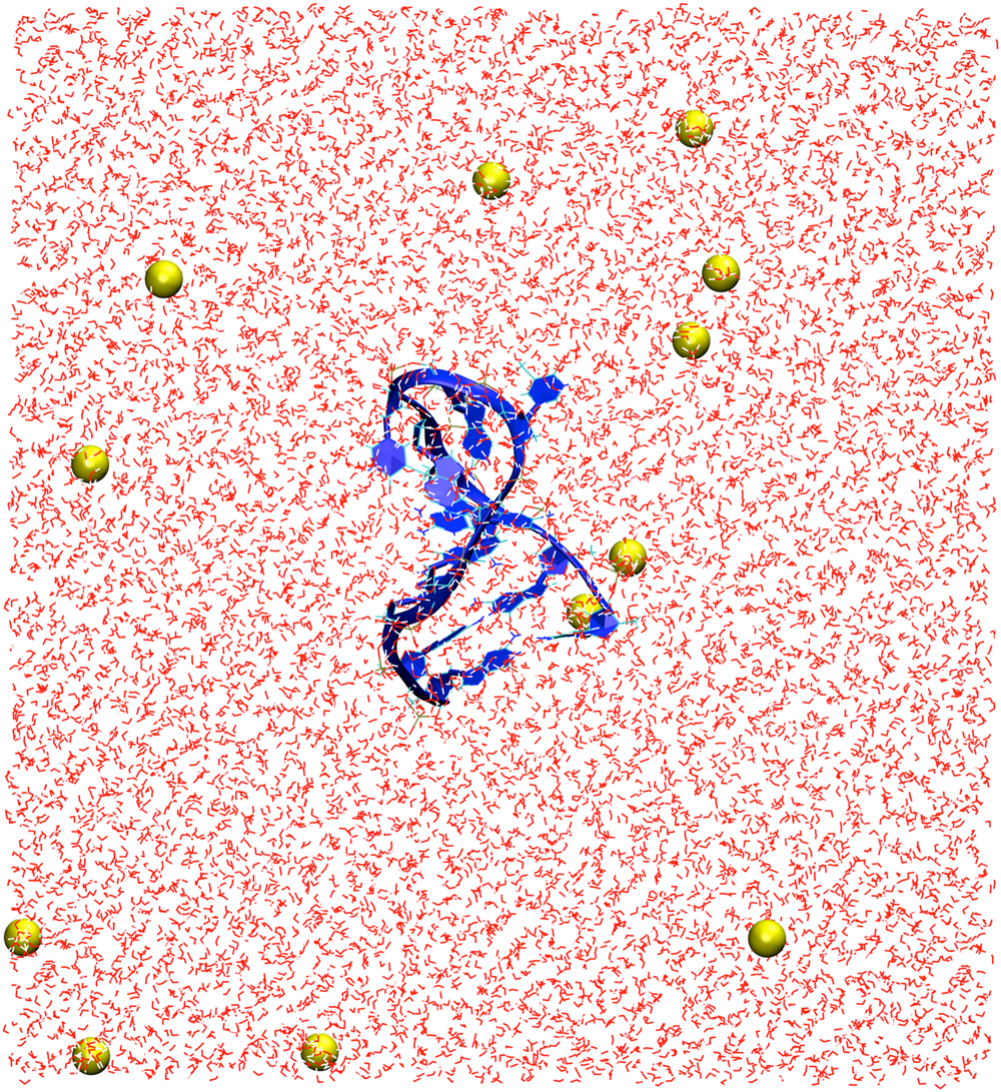
Initial configuration of the MD simulation of the predicted 3D ssDNA structure for the sequence CGCGGTGTCCGCG, corresponding to 1LA8. Only the molecules within the central simulation cell are shown. The ssDNA molecule was solvated in water and the system charge neutralized with 12 sodium ions. The atoms of the water molecules are represented as white (hydrogen) and red (oxygen) lines and the sodium ions are displayed as yellow spheres with radii corresponding to its atomic van der Waals radius. The ssDNA molecule is shown in blue with the backbone and bases represented as new ribbons and the additional components represented as lines. The unit cell dimensions are *L*_x_ = 72 Å, *L*_y_ = 72 Å, and *L*_z_ = 78 Å.

**Figure 5:**
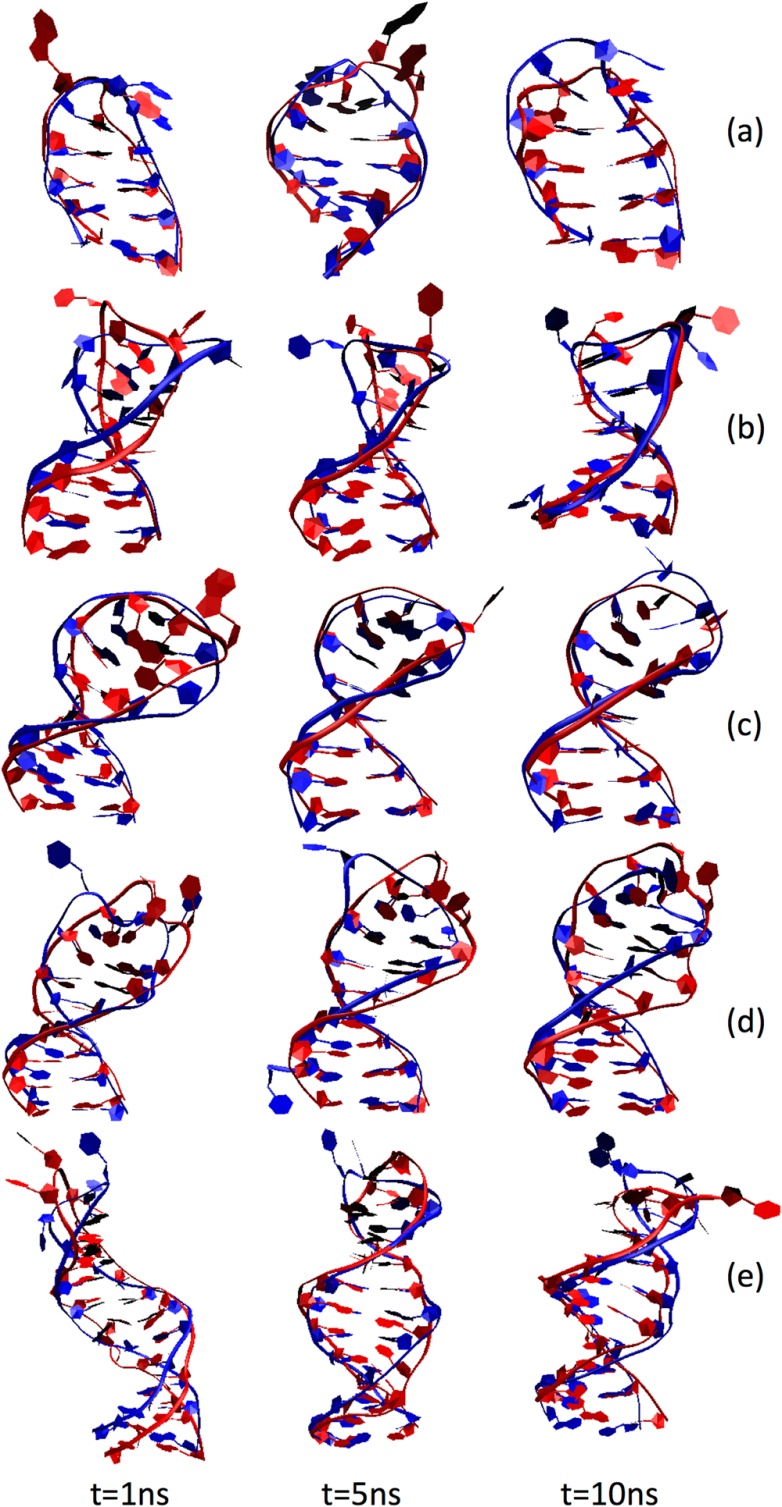
Evolution of the ssDNA molecules as a function of time. The overlays of the predicted structures taken from the MD simulations (ssDNA colored red) and the corresponding experimental structures downloaded from the PDB database (ssDNA colored blue) correspond to (**a**) 1BJH, (**b**) 1LA8, (**c**) 2M8Y, (**d**) 2VAH, and (**e**) 2L5K. The snapshots for the predicted ssDNA molecules correspond to (from *left* to *right*) 1 ns, 5 ns, and 10 ns. The corresponding RMSD values are summarized in Table 3. Water molecules and ions within the simulation cell are not shown.

We have used fully atomistic molecular dynamics (MD) simulations to further improve our structural predictions for the 1BJH, 1LA8, 2M8Y, 2VAH, and 2L5K cases and study the dynamics of the systems. For these cases, the 3D structural predictions obtained from sequence (Fig. 2) were solvated in water and used as initial configurations of the molecular dynamics simulations, as detailed in the methods section. Figure 4 shows the initial configuration of the MD simulation corresponding to the 1LA8 ssDNA structure, solvated in water containing 12 sodium ions. In all five cases, the DNA structures evolve as a function of time during the 10-ns length of the simulations. In Fig. 5, we display three snapshots for each of the five different ssDNAs obtained from the simulations at different time intervals, which correspond to 1 ns, 5 ns, and 10 ns. Our results indicate that, in all cases, the hairpin structure of the ssDNAs remains stable during the 10 ns of the simulations. The overlays of Fig. 5 correspond to the ssDNA structures at the different time intervals (ssDNA colored red) with respect to the corresponding structure downloaded from the PDB database (ssDNA colored blue). The corresponding RMSD values calculated at different times along the 10-ns simulation, corresponding to 1 ns, 2 ns, 3 ns, 4 ns, 5 ns, 6 ns, 7 ns, 8 ns, and 9 ns, are summarized in Table 3. These values are typical and in the same range as values used to demonstrate similarity of proteins^51, 52^. The quantitative RMSD calculation and the qualitative visual comparison of the pairs of structures show that the approach successfully predicts the three-dimensional structure of ssDNA hairpins.

**Table 3:** RMSD values (in Angstroms) of the sugar-phosphate backbone for the predicted ssDNA structures at different time points along the MD simulations with respect to the corresponding experimental structures downloaded from the PDB database.

	1BJH	1LA8	2M8Y	2VAH	2L5K
3D-Prediction	2.41	3.98	3.56	4.10	4.07
1 ns	2.00	3.44	1.46	3.16	7.02
2 ns	4.40	4.28	1.56	3.06	5.77
3 ns	2.11	2.73	2.04	3.26	4.69
4 ns	2.36	3.69	2.05	3.36	4.69
5 ns	2.17	2.95	1.62	3.07	2.79
6 ns	2.47	2.92	1.35	3.53	2.90
7 ns	3.22	1.78	1.65	2.93	3.59
8 ns	2.64	2.28	2.90	2.84	3.12
9 ns	3.32	2.32	1.85	2.69	4.32

The corresponding values for the 3D predictions (Fig. 2) are included for completeness.

**Figure 6:**
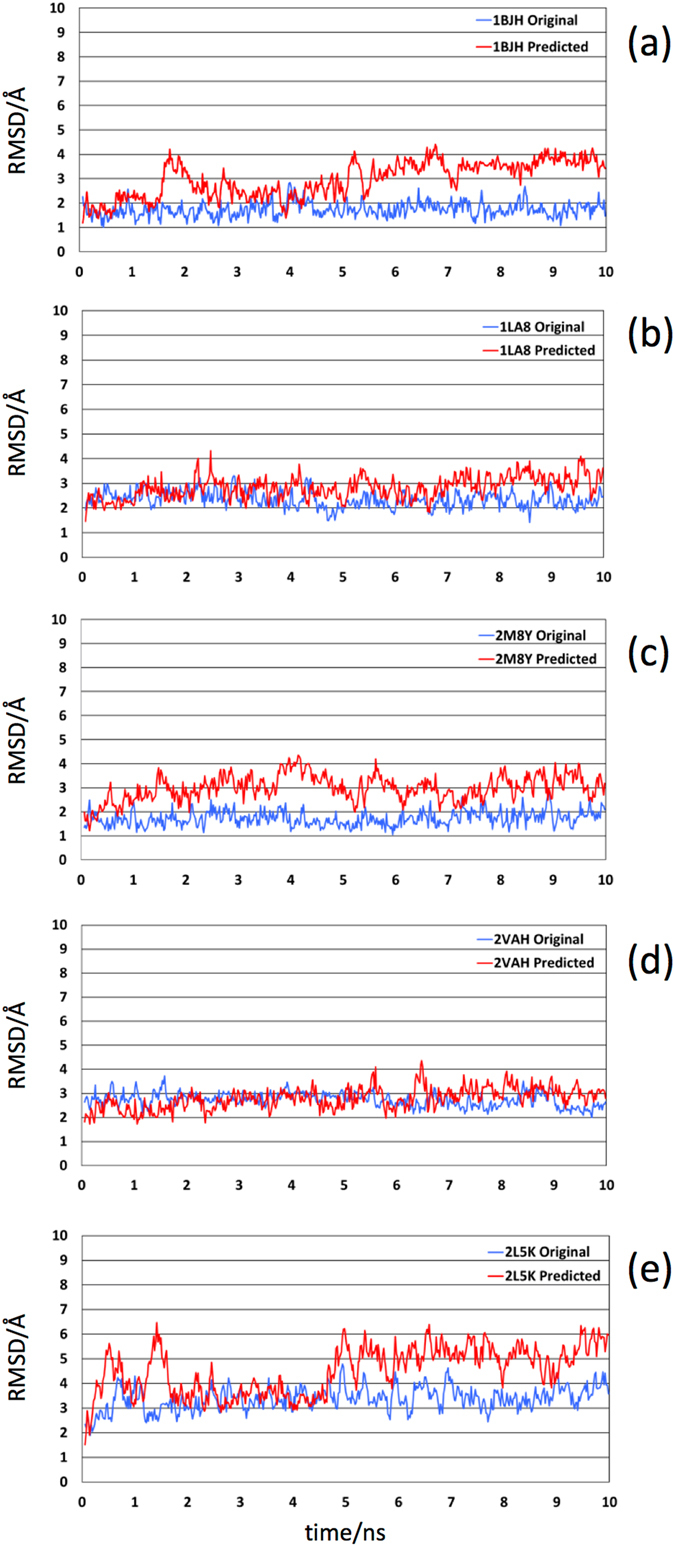
Time evolution of the RMSDs for the sugar-phosphate backbone with respect to the structures at time 0 for the predicted (red curves) and original (blue curves) structures. The panels correspond to (**a**) 1BJH, (**b**) 1LA8, (**c**) 2M8Y, (**d**) 2VAH, and (**e**) 2L5K.

Similar MD simulations were performed using the experimentally resolved structures as initial configurations, following the approach detailed in the methods section. To explore the structural deviation of the ssDNA molecules with respect to their corresponding starting structure, the RMSD of the sugar-phosphate backbone of each structure was calculated independently over the course of the 10-ns trajectory. The RMSDs provides valuable information on the conformational flexibility of the biomolecule and provides a means to evaluate the structural deviation of the biomolecule through time. The results of the RMSD studies (Fig. 6) show that the predicted structures have similar time-dependent behavior in comparison to the original structures.

## Conclusions

DNA aptamers are more stable than their RNA counterparts, which is especially relevant for biomedical applications, but lack the wide range of computational tools for structural predictions currently available for single stranded RNA. Here, we have presented the first approach to predict the prototypical 3D structures of ssDNA required for aptamer applications. So far, there has not been a computational tool available for 3D structure prediction of DNA aptamers from their sequence and very few structures have been resolved experimentally. These limitations have constituted a major bottleneck for the emergent application of aptamers in biotechnology and for clinical use in biomedical applications. Our results strongly support that it is possible to accurately determine the structure of single-stranded DNA from sequence and provide an approach that works exceptionally well for the hairpin-like structural motif.

We have shown that our approach faithfully predicts representative structures available in the Protein Data Bank and Nucleic Acids databases. Specifically, we have extensively validated the prediction capabilities of our approach against a pool of 24 ssDNA molecules with experimentally solved 3D structures selected through an exhaustive search for ssDNA molecules and aptamers in the PDB database (http://www.pdb.org), with sequences ranging from 7 to 27 nucleotides. The studied cases are representative of a wide variety of systems of biological and biomedical interest, including Mucin 1-binding aptamers and ssDNAs with uracil bases. The resulting structures can subsequently be used as inputs in computational docking methods to study the interactions with ligands^53^.

We have shown that atomistic MD simulations can be used to further improve the structural predictions by studying the dynamics of the systems under conditions that mimic their targeted environment. Our results indicate that our approach works exceptionally well for the most common hairpin-like structural motif of ssDNA and it is expected to work for other systems with similar interactions between bases, like bulge loops and internal loops. Our results break the ground for future work to expand the applicability of our approach to other ssDNA folds, including G-quadruplex folds, typical of G-rich sequences such as those in the thrombin-binding aptamer, as well as the effect of covalent modifications to increase the stability of aptamers. For instance, it has been shown that phosphorothioate substitutions can substantially alter RNA conformation^54^.

Understanding the specific interactions involved in stabilizing biomolecular complexes immobilized on solid substrates is essential for designing biosensors with improved sensitivity, specificity, and reliability. Several key studies have shown that the sensitivity of aptamer-based biosensors is related to the surface crowding of the immobilized aptamers on the biosensor surface^11, 55, 56^ and have suggested that steric hindrance or electrostatic repulsion of the charged aptamer molecules at higher concentrations on the surface of the biosensor have an impact on biosensor performance. However, this area is still poorly understood because of the lack of understanding of the molecular level interactions occurring at the biosensor surface. Furthermore, understanding how small sequence changes can lead to a difference in affinity can be used to make marked improvements in biosensor performance by employing rational design techniques^57–60^. Our approach, therefore, provides a much-needed starting point to gain better insights in the performance of aptamer-based biosensors with the aim of improving biosensor performance by rational design techniques.

## Electronic supplementary material

Supplementary Information
