# Supplementary material for: Three-dimensional modeling of single stranded DNA hairpins for aptamer-based biosensors
Source: Sci Rep. 2017 Apr 26;7:1178. doi: 10.1038/s41598-017-01348-5 (PMC5430850; doi:10.1038/s41598-017-01348-5)
Supplement: Supplementary file 1 — Supplementary Information [file 41598_2017_1348_MOESM1_ESM.pdf]

Three-dimensional modeling of single stranded DNA hairpins for aptamer-based biosensors

Iman Jeddi[1] and Leonor Saiz[1,*]

[1]Modeling of Biological Networks and Systems Therapeutics Laboratory, Department of Biomedical Engineering, University of California, 451 East Health Sciences Drive, Davis, CA 95616, USA.

Corresponding author:
Leonor Saiz
Department of Biomedical Engineering
University of California
451 E. Health Sciences Drive, Davis, CA 95616, USA
Tel: (530) 752-6700
Fax: (530) 754-5739
Email: lsaiz@ucdavis.edu

Keywords: aptamer, hairpin, single stranded DNA, computational modeling, biosensor

* To whom correspondence should be addressed: lsaiz@ucdavis.edu

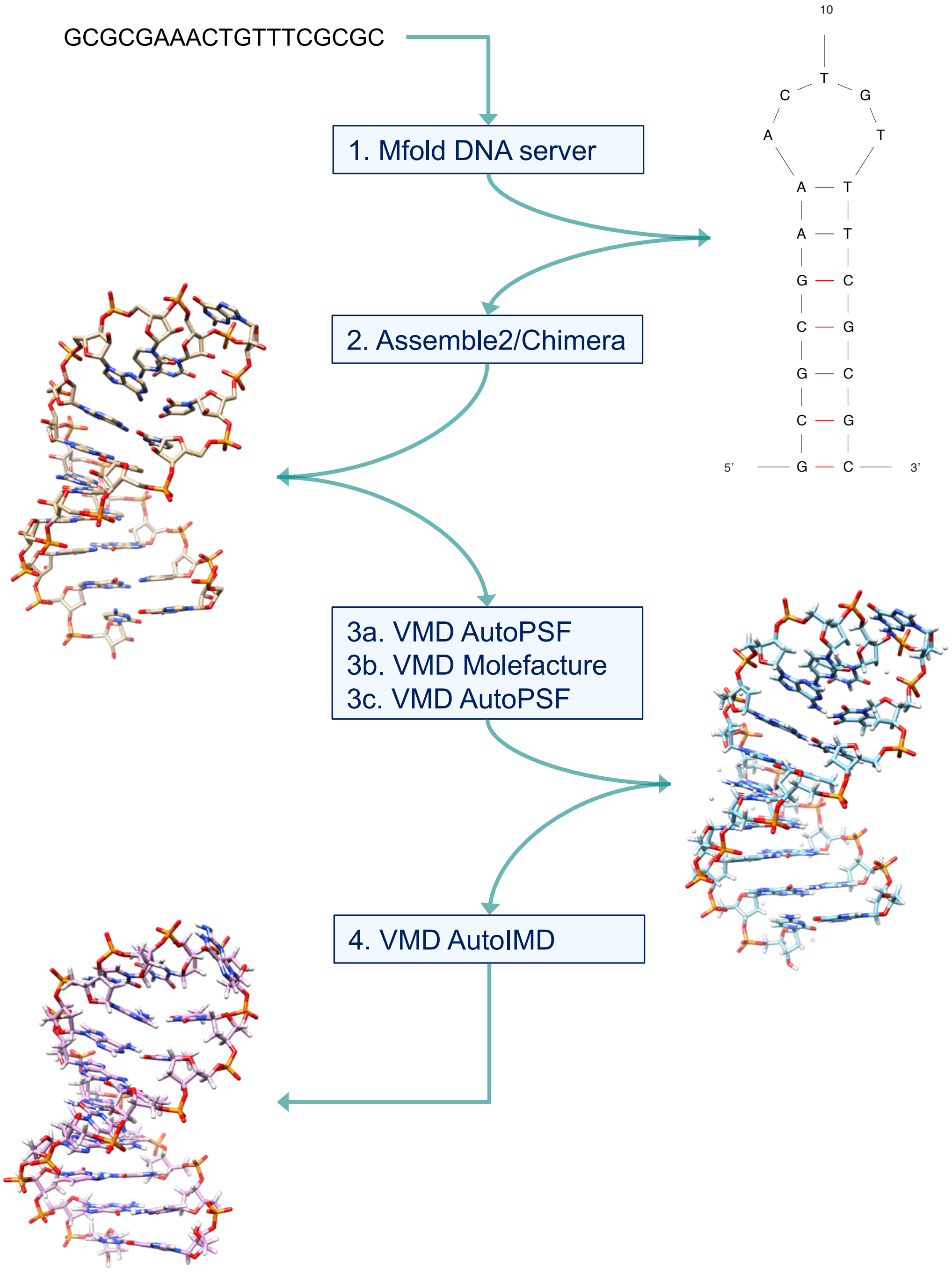

Supplementary Figure 1: Structures obtained at the end of each of the four steps of the workflow for the sequence GCGCGAAACTGTTTCGCGC of the structure with PBD ID 1ECU.
